# Preliminary Evaluation of the Diagnostic Performance of OvMANE1 and OvMCBL02 Multiepitope Antigens for the Non-Invasive Diagnosis of Onchocerciasis Exposure

**DOI:** 10.3390/life15101515

**Published:** 2025-09-26

**Authors:** Bernis Neneyoh Yengo, Cabirou Mounchili Shintouo, Robert Adamu Shey, Ntang Emmaculate Yaah, Luc Vanhamme, Rose Njemini, Jacob Souopgui, Stephen Mbigha Ghogomu

**Affiliations:** 1 Department of Microbiology and Immunology, College of Medicine, Drexel University, 2900 W Queen Ln, Philadelphia, PA 19129, USA; by339@drexel.edu; 2 Department of Biochemistry and Molecular Biology, Faculty of Science, University of Buea, Buea P.O. Box 63, Cameroon; sheynce@gmail.com (R.A.S.); mayaahemma@gmail.com (N.E.Y.); 3 Department of Molecular Biology, Institute of Biology and Molecular Medicine, IBMM, Université Libre de Bruxelles, Gosselies Campus, 12, 6040 Gosselies, Belgium; luc.vanhamme@ulb.be (L.V.); jacob.souopgui@ulb.be (J.S.); 4 Department of Gerontology, Faculty of Medicine and Pharmacy, Vrije Universiteit Brussel, Laarbeeklaan 103, 1090 Brussels, Belgium; rose.njemini@vub.be; 5 Frailty & Resilience in Ageing Research Unit, Vitality Research Group, Vrije Universiteit Brussel, Laarbeeklaan 103, 1090 Brussels, Belgium

**Keywords:** onchocerciasis, diagnosis, OvMANE1, OvMCBL02, urine, non-invasive

## Abstract

A shift in the public health goal for onchocerciasis from control to elimination implies that the treatment of onchocerciasis must be extended to communities that are hypoendemic for the disease. However, in such communities, the majority of the population may not manifest the symptoms of onchocerciasis. As a result, they may be reluctant to take part in epidemiological surveys aimed at monitoring parasite transmission, particularly due to the invasive nature of the currently approved diagnostic tests. This reluctance is compounded by the absence of visible, severe manifestations of the disease in these areas. On the other hand, diagnostic methods that utilize samples collected by a non-invasive procedure, such as urine, are generally painless and not risky. In this context, we evaluated the diagnostic performances of OvMANE1 and OvMCBL02 multiepitope antigens using urine samples. The evaluation of total IgG and IgG subclass responses revealed IgG3 as the most effective IgG for the OvMANE1 test (sensitivity = 87.5%, specificity = 100.0%), total IgG for the OvMCBL02 test (sensitivity = 92.5%, specificity = 100.0%), and IgG3 for the OvMANE1_OvMCBL02 cocktail test (sensitivity = 92.5%, specificity = 100.0%). These tests have the potential to meet the criteria of a diagnostic test’s target product profile to map onchocerciasis in low-prevalence areas, where a sensitivity of ≥60.0% and specificity of ≥99.8% are recommended. Furthermore, the OvMCBL02 and OvMANE1_OvMCBL02 cocktail tests may have the features of a diagnostic test’s target product profile to determine treatment endpoints (recommended sensitivity ≥ 89.0%, specificity ≥ 99.8%) as reported by the Diagnostics Technical Advisory Group for Neglected Tropical Diseases of the World Health Organization. Consequently, further characterization of these multiepitope antigens may enable urine, which can be collected non-invasively, to be used in the OvMANE1 and OvMCBL02 tests for the field evaluation of onchocerciasis.

## 1. Introduction

Onchocerciasis is classified by the World Health Organization (WHO) as a “neglected tropical disease (NTD)” caused by infection with a filaria nematode known as *Onchocerca volvulus* (*O. volvulus*) [1]. The pathology of onchocerciasis primarily arises from the inflammation triggered by dying microfilariae in various parts of the body. In the skin, this inflammation leads to symptoms such as intense itching and reactive skin conditions, and over time can result in skin discoloration, thinning, and the development of hanging groin. When inflammation occurs in the eyes, it can cause corneal lesions, which if left untreated can result in blindness [2]. The 2021–2030 blueprint for NTDs, created to end neglect and contribute to achieving the Sustainable Development Goals, was formally endorsed in 2021 by the WHO [3]. The public health goal of onchocerciasis within this blueprint is elimination. Although multifactorial, the availability of an accurate, non-invasive, and easy-to-use diagnostic test for onchocerciasis is essential to achieving the elimination of the disease by 2030 [4,5]. Microscopic analysis of surgically removed onchocercal nodules can be used to assess the viability of *O. volvulus* adult worms; however, this method is invasive, costly, requires specialized skills, and is not practical for large-scale surveillance or in resource-limited settings [6]. The Ov16 antigen-based ELISA, which detects exposure to *Onchocerca volvulus* infection, remains a key diagnostic tool in onchocerciasis surveillance. However, its lack of standardization and limited adaptability for point-of-care restricts its utility in endemic settings [7].

Currently, the skin snip test, which detects *O. volvulus* microfilariae in skin biopsies under a microscope, is the gold standard test for onchocerciasis [8]. However, it has been criticized for its inability to detect infections with low microfilaria loads and those with pre-patent infections [9,10]. Furthermore, the procedure is invasive and carries a threat of infection [11,12]. Hence, it may be difficult to conduct field evaluations of onchocerciasis prevalence in a community due to the low sensitivity of the skin snip test and the population’s aversion to being subjected to repeated invasive tests during the monitoring phase of an onchocerciasis treatment plan [13]. These limitations underscore the urgent need for novel, highly sensitive, and field-deployable diagnostic platforms to advance onchocerciasis elimination strategies.

On the other hand, diagnosis using urine samples is usually painless, more practical, affordable, and not risky. Urine can often be provided promptly upon request, and the procedure for urine collection is non-invasive. Furthermore, urine is easier to manage in terms of collection, storage, and handling, and it is more acceptable to patients [14]. Aside from these benefits, several urine-based diagnostic tests have also been found to be more sensitive than conventional techniques that are invasive, especially in areas of low endemicity [15,16]. Additionally, antibody-detection ELISA using urine samples has been used to identify disease outbreaks in their early stages [17] and to assess disease severity [18]. Western and dot blot assays have been used for the detection of *Onchocerca volvulus* heat shock protein 70 (*Ov*HSP70) in the urine of individuals infected with onchocerciasis [19]. In recent studies, the multiepitope antigens OvMANE1 [20] and OvMCBL02 [21] have been designed and validated as potential biomarkers for the identification of *O. volvulus* infection. Consequently, the diagnostic performance of these potential biomarkers using urine samples has been evaluated for potential use in the field-based assessment of *O. volvulus* infection.

## 2. Materials and Methods

### 2.1. Ethical Considerations

This work received ethical approval from the Cameroon Bioethics Initiative (CAMBIN) Ethics Review and Consultancy Committee (ERCC) (approval number: CBI/443/ERCC/CAMBIN). All individuals who participated in the study did so voluntarily. Following detailed descriptions of what the project involved, the participants signed the informed consent forms. The privacy of the participants was safeguarded throughout the data collection, processing, and reporting.

### 2.2. Study Site, Population, and Sample Collection

Before the collection of urine samples, a trained and certified medical practitioner conducted a clinical examination for signs of onchocerciasis among individuals who accepted taking part in the study from the Massangam Health District in the West Region of Cameroon. A microscopy-based *O. volvulus* identification in skin snips was performed using a sterile Holtz corneo-scleral punch to collect two skin snips from each participant’s left and right iliac crests. The skin biopsies were transferred into separate wells of a microtiter plate, and a few drops of saline were added before being cultured for 24 h to enable microfilariae to emerge from the snips. The presence of microfilariae in the skin snips was determined with the aid of an inverted microscope. Individuals infected with *O. volvulus* were selected based on the existence of clinical manifestations of onchocerciasis and the detection of microfilaria in skin biopsies. Control urine samples were collected from healthy Cameroonians living in Belgium, a non-endemic zone, who had not visited an onchocerciasis endemic area for at least 5 years. The control participants did not present any sign of the clinical manifestation of onchocerciasis and had not received ivermectin treatment for a minimum of 5 years. Urine samples from *Schistosoma haematobium*-infected individuals were collected from persons who were tested by skin snip microscopy to be negative for *O. volvulus* in Tiko, Cameroon’s Southwest Region. These samples were used to test for cross-reactivity of the chimeric antigens.

### 2.3. Enzyme-Linked Immunosorbent Assay (ELISA)

The antibody-capture ELISA was performed according to the method previously described by Ghosh et al. [22] with slight modifications. Briefly, MaxiSorp 96-well microtiter plates (Nunc, Roskilde, Denmark) were coated with 50 µL of 2 µg/mL of the OvMANE1, OvMCBL02, or OvMANE1_OvMCBL02 cocktail multiepitope antigen in carbonate buffer, pH 9.6, and incubated for 2 h at room temperature. The plates were washed three times at 5 min intervals with wash buffer (PBS containing 0.05% Tween 20; pH 7.4) and then incubated overnight with blocking buffer (PBST containing 1% BSA; pH 7.4) at 4 °C. On the following day, the plates were washed 3 times with the wash buffer. Seven different dilutions (1:5, 1:10, 1:20, 1:40, 1:80, 1:160, 1:320) along with undiluted urine were initially tested.

Finally, the optimally diluted urine in PBS containing 0.1% BSA and 0.05% Tween 20 was applied to the respective wells and then incubated for 90 min at room temperature. The plates were washed 3 times with wash buffer, and then 50 µL of goat anti-human IgG (Fc-specific) peroxidase conjugate (1:2000) (Sigma, St. Louis, MO, USA) was added to each well and incubated for 90 min at room temperature. For the IgG subclass ELISA, the urine incubation step was followed by a 90-min incubation with mouse monoclonal anti-human IgG1, IgG2, IgG3, and IgG4 Fc (HRP) antibody (1:2000) (Sigma, St. Louis, MO, USA) as the secondary antibody. After washing as above, conjugated anti-mouse IgG, an HRP-link antibody produced in rabbit (1:5000) (Sigma, St. Louis, MO, USA), was used as a tertiary antibody in the case of IgG1, IgG2, and IgG3. After the last washing step, 50 μL of TMB substrate (Sigma, St. Louis, MO, USA) was added to each well, and the plates were incubated at room temperature for 5 min. After stopping the reactions with 1 M hydrochloric acid, the optical densities were measured at 450 nm.

### 2.4. Data Analysis

Microsoft Excel 365 was used for the analysis of the data collected for this study. The normality of distributions was determined using the Shapiro–Wilk test, and GraphPad Prism 9.3.1 (La Jolla, CA, USA) was used to generate scatter plots. For multiple comparisons among the three groups, Dunn’s test was employed after the Kruskal–Wallis test. The ability of total IgG to discriminate was assessed using receiver operating curve analysis. The area under the receiver operating curve was computed using the trapezoid technique, and an optimal cutoff value was chosen based on Youden’s index to estimate the sensitivities, specificities, and 95% confidence intervals for the chosen cutoff value. False negatives (FNs) were calculated using sensitivity and the number of actual positive cases (P), based on the formula FN = P × (1 − Sensitivity), while false positives (FPs) were determined using specificity and the number of actual negative cases (N), following the formula FP = N × (1 − Specificity). At a *p*-value of 0.05 and below, all results were deemed statistically significant.

## 3. Results

### Antibody Capture ELISA to Determine Exposure of O. volvulus Infection

In a bid to develop a non-invasive test that can be used for the field evaluation of onchocerciasis in the era of onchocerciasis elimination, urine from (i) actively *O. volvulus*-infected individuals (OVU, *n* = 40), (ii) healthy Cameroonians living in Belgium without visiting onchocerciasis endemic regions for at least 5 years (HEU, *n* = 23), and (iii) *S. haematobium*-infected individuals (SHU, *n* = 7) were analyzed for the presence of OvMANE1 and OvMCBL02 specific antibodies. A 1:5 dilution was selected as the optimal dilution of urine for the antibody ELISA using a 10-man pool of *O. volvulus*-infected (OVU) and control (HEU) urine samples.

**Figure 1:**
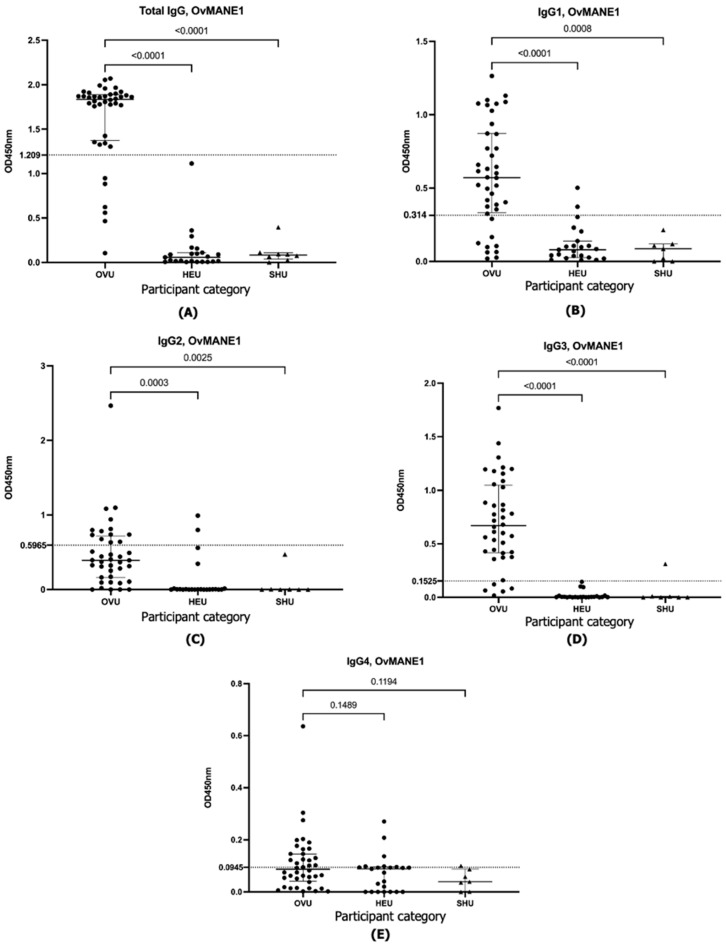
Exposure of humans to parasite transmission leads to increased OvMANE1-specific total IgG, IgG1, and IgG3 in urine from infected but not from uninfected controls. Microtiter plates were coated with purified OvMANE1 chimeric antigen. Total IgG (**A**), IgG1 (**B**), IgG2 (**C**), IgG3 (**D**), and IgG4 (**E**) responses were measured in all of the urine samples diluted 1:5. For total IgG and IgG4, HRP-conjugated mouse anti-human antibodies were added, while for IgG1, IgG2, and IgG3 measurements, the HRP-conjugated anti-mouse antibody was used. OVU = urine from *O. volvulus*-infected individuals (*n* = 40); HEU = urine from healthy Cameroonian controls (*n* = 23), and SHU = urine from schistosomiasis patients (*n* = 7). A Kruskal–Wallis test followed by Dunn’s test was used for multiple comparisons.

**Figure 2:**
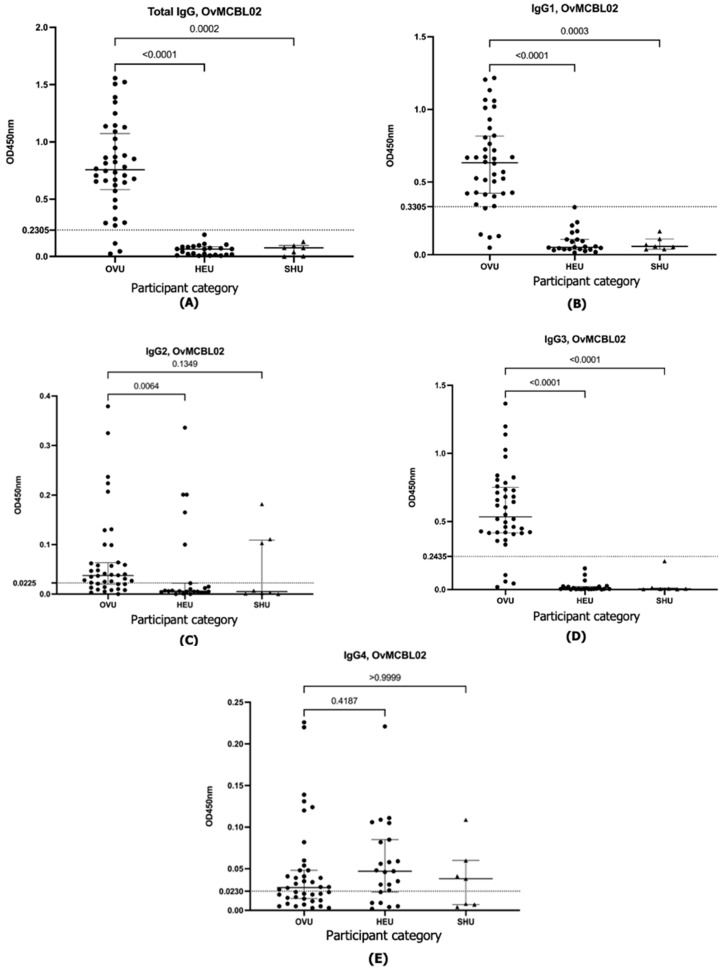
Exposure of humans to parasite transmission leads to increased OvMCBL02-specific total IgG, IgG1, and IgG3 in urine from infected but not from uninfected controls. Microtiter plates were coated with the purified OvMCBL02 multiepitope antigen. Total IgG (**A**), IgG1 (**B**), IgG2 (**C**), IgG3 (**D**), and IgG4 (**E**) responses were measured in all of the urine samples diluted 1:5. For total IgG and IgG4, HRP-conjugated mouse anti-human antibodies were added, while for IgG1, IgG2, and IgG3 measurements, HRP-conjugated anti-mouse antibody was used. OVU = urine from *O. volvulus*-infected individuals (*n* = 40); HEU = urine from healthy Cameroonian controls (*n* = 23), and SHU = urine from schistosomiasis patients (*n* = 7). A Kruskal–Wallis test followed by Dunn’s test was used for multiple comparisons.

**Figure 3:**
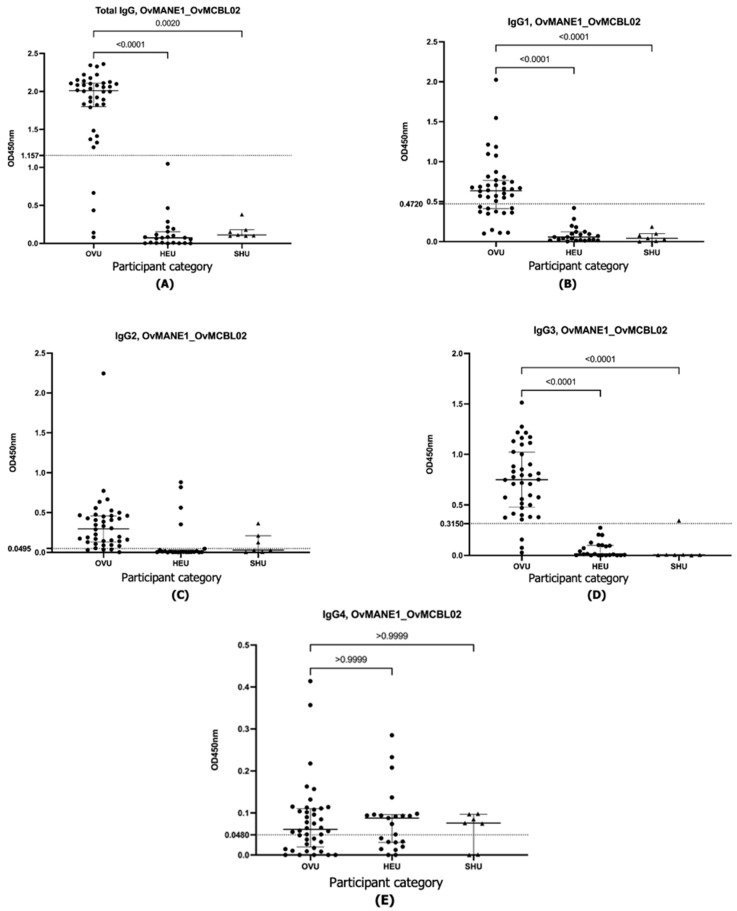
Exposure of humans to parasite transmission leads to an increase in a cocktail of OvMANE1_OvMCBL02-specific total IgG, IgG1, and IgG3 in urine from infected but not from uninfected controls. Microtiter plates were coated with the purified OvMANE1_OvMCBL02 cocktail multiepitope antigen. Total IgG (**A**), IgG1 (**B**), IgG2 (**C**), IgG3 (**D**), and IgG4 (**E**) responses were measured in all of the urine samples diluted 1:5. For total IgG and IgG4, HRP-conjugated mouse anti-human antibodies were added, while for IgG1, IgG2, and IgG3 measurements, HRP-conjugated anti-mouse antibody was used. OVU = urine from O. volvulus-infected individuals (*n* = 40); HEU = urine from healthy Cameroonian controls (*n* = 23), and SHU = urine from schistosomiasis patients (*n* = 7). A Kruskal–Wallis test followed by Dunn’s test was used for multiple comparisons.

The results obtained revealed that both the OvMANE1 and OvMCBL02 tests efficiently discriminated between infected and uninfected urine samples when the multiepitope antigens reacted with total IgG, IgG1, and IgG3 (*p* < 0.0001) (Figure 1, Figure 2 and Figure 3). When the multiepitope antigen reacted with IgG2 and IgG4, the tests were unable to distinguish between infected and uninfected urine samples. Diagnostic accuracy parameters investigated for the IgG and IgG subclass responses revealed IgG3 as the favorite for the OvMANE1 and OvMANE1_OvMCBL02 cocktail tests and total IgG for the OvMCBL02 test (Table 1).

## 4. Discussion

The present investigation led to the development of two non-invasive, sensitive, and accurate tests for exposure to *O. volvulus* infection using the multiepitope antigens OvMANE1 and OVMCBL02 to detect the total IgG and IgG3 antibodies in the patients’ urine. To the best of our knowledge, these test configurations are reported herein for the first time, and if confirmed in larger studies may serve to monitor onchocerciasis elimination campaigns, especially in hypoendemic areas. Despite antibody levels in urine being roughly 4000 to 10,000 times lower than in serum [23], antibodies can still be quantitatively detected using a urine-based ELISA method [24]. Indeed, multiple urine-based ELISA techniques have identified pathogen-specific IgG including its various subclasses, which are the most prevalent antibodies in circulation [14,25].

In the search for a non-invasive test for onchocerciasis using urine samples, OvMANE1 and OvMCBL02 multiepitope antigens, which have previously been reported to be potential diagnostic biomarkers for onchocerciasis diagnosis based on serum samples [20,21], were evaluated in this study to determine their diagnostic performance using urine samples from onchocerciasis patients in Cameroon, healthy Cameroonians living in Belgium as well as schistosomiasis patients. A survey of the geographic distribution of onchocerciasis in Cameroon found that the disease was endemic across all ten regions, with about 60% of the population living in high-risk areas [26]. Despite nearly two decades of mass preventive chemotherapy in endemic communities, ongoing transmission has been observed [27,28]. Since the OvMANE1 and OvMCBL02 tests detect antibodies and cannot distinguish between past and current infections, control samples were sourced from healthy Cameroonians residing in Belgium who had not traveled to onchocerciasis-endemic areas for at least five years. This approach was taken to minimize the risk of including individuals who may have been previously exposed to the parasite, which could have been the case with controls sourced from within Cameroon.

The multiepitope antigens discriminated between urine from infected and uninfected individuals. Following prioritization of the specificity of the tests, a cutoff value based on Youden’s index was chosen to determine the corresponding sensitivity of the test. Diagnostic performance evaluated for the total IgG and IgG subclass responses revealed IgG3 as the favorite for the OvMANE1 test (sensitivity = 87.5%, specificity = 100.0%), total IgG for the OvMCBL02 test (sensitivity = 92.5%, specificity = 100.0%), and IgG3 for the OvMANE1_OvMCBL02 cocktail test (sensitivity = 92.5%, specificity = 100.0%). Although the sensitivity of the OvMANE1 and OvMCBL02 tests when reacted with serum samples was comparable (98.08% and 98.40%, respectively) [20,21], the sensitivity of the OvMCBL02 test was higher in the current study when reacted with urine samples. Also, a cocktail of both multiepitope antigens did not result in a higher sensitivity than that of the OvMCBL02 test. Hence, the OvMCBL02 multiepitope antigen may be a better biomarker than OvMANE1 to determine the exposure of *O. volvulus* infection using urine samples. However, both tests have the potential to meet the diagnostic performance criteria of the target product profile of a diagnostic test to map onchocerciasis in low prevalence areas (recommended sensitivity 60.0%, specificity 99.8%). Additionally, the OvMCBL02 and OvMANE1_OvMCBL02 cocktail tests may possess the features of the target product profile of a diagnostic test to determine when to stop treatment with ivermectin (recommended sensitivity ≥89.0%, specificity ≥99.8%) [29]. Although the OvMANE1 and OvMCBL02 assays exhibit comparable sensitivity and specificity when performed on urine and serum samples [8,21], it is important to highlight that the dilution factor of the urine was 5, which is lower than that of serum at 2000. With this urine dilution factor of 5, the titer of antibodies was high enough for effective detection. This difference reflects the substantially lower concentration of antibodies in urine, which can be up to 10,000 times lower than in serum.

The humoral immune response of IgG4 to the OvMANE1 and OvMCBL02 multiepitope antigens could not discriminate between urine samples from the infected and uninfected individuals. This is consistent with the results obtained when the multiepitope antigens were reacted with pooled serum samples, as the IgG4 levels were low or absent in response to the different multiepitope antigens [20,21]. When the multiepitope antigens were reacted with urine samples from Schistosomiasis patients, no non-specific reactions were observed. Although the sample size of *S. haematobium* infected individuals was small (*n* = 7), these multiepitope antigens did not also cross-react with serum from individuals infected with related nematode species in our previous studies [8,21]. This contrasts with our findings using an *O. volvulus* excretory-secretory product Ov28CRP/OvGM2AP, which did exhibit cross-reactivity with the same sample set [30]. Similarly, other authors have reported the cross-reactivity of *O. volvulus* antigens with that of other nematode parasites in non-onchocerciasis endemic regions [31]. Although a *cDNA* clone of *O. volvulus* encoding a microfilarial surface-associated antigen did not cross-react with samples from individuals infected with *Brugia malayi* or *Loa loa*, it was deemed unsuitable as a diagnostic biomarker for onchocerciasis due to its reactivity with samples from *Wuchereria bancrofti* infections [32].

Antibody-based tests present several challenges. Individuals who have previously cleared the infection may still test positive due to lingering antibodies, resulting in false-positive outcomes. Moreover, because antibody production takes time, early stages of infection may not be detected, since exposure to *O. volvulus* needs time for antibodies to be generated. The reliability of antibody-based tests is further reduced in immunocompromised individuals, who may have a diminished antibody response. As a result of these limitations, there has been a growing shift toward antigen-based diagnostics, which offer a more accurate indication of active infection.

Conclusively, the OvMANE1 and OvMCBL02 tests demonstrated promising potential as diagnostic tools for the field evaluation of *O. volvulus* transmission using urine samples. This is only the first part of the study analyzing the ability to detect the exposure of specific antibodies in urine. To further validate their utility, additional characterization of these multiepitope antigens is warranted using a larger set of urine samples from individuals infected with *O. volvulus*, *Wuchereria*, *L. loa*, and *Mansonella* species. This will enable the analysis of possible cross reactions with other parasites. Such efforts may support the development of a reliable, non-invasive diagnostic approach for onchocerciasis, leveraging urine as an accessible and patient-friendly biological fluid.

## Figures and Tables

**Table 1:** Receiver operating curve (ROC) values and diagnostic accuracy parameters for total IgG and IgG subclass responses to the OvMANE1, OvMCBL02, and OvMANE1_OvMCBL02 cocktail multiepitope antigens using urine samples.

OvMANE1		Total IgG	IgG1	IgG2	IgG3	IgG4
ROC analysis	ROC area (AUC)	0.9880	0.8864	0.8000	0.9853	0.6321
	95% CI of AUC	0.9687 to 1.000	0.8063 to 0.9665	0.6706 to 0.9294	0.9645 to 1.000	0.4853 to 0.7788
	*p*-value (against AUC = 0.5)	<0.0001	<0.0001	<0.0001	<0.0001	0.0828
Diagnostic accuracy parameter	Cutoff value	1.209	0.3140	0.5965	0.1525	0.0945
	Sensitivity (%) (95% CI)	85.0 (70.93% to 92.94%)	77.5 (62.50% to 87.68%)	32.5 (20.08% to 47.98%)	87.5 (73.89% to 94.54%)	47.5 (32.94% to 62.50%)
	Specificity (%) (95% CI)	100.0 (85.69% to 100.0%)	91.3 (73.20% to 98.45%)	91.30 (73.20% to 98.45%)	100.0 (85.69% to 100.0%)	73.9 (53.53% to 87.45%)
	False negative	15.0%	22.5%	67.5%	12.5%	52.5%
	False positive	0.0%	8.7%	8.7%	0.0%	26.1%
**OvMCBL02**						
ROC analysis	ROC area (AUC)	0.9707	0.9685	0.7391	0.9837	0.5973
	95% CI of AUC	0.9304 to 1.000	0.9315 to 1.000	0.5902 to 0.8881	0.9612 to 1.000	0.4465 to 0.7481
	*p*-value (against AUC = 0.5)	<0.0001	<0.0001	0.0017	<0.0001	0.2014
Diagnostic accuracy parameter	Cutoff value	0.2305	0.3305	0.0225	0.2435	0.0230
	Sensitivity (%) (95% CI)	92.5 (80.14% to 97.42%)	87.5 (73.89% to 94.54%)	70.0% (54.57% to 81.93%)	90.0 (76.95% to 96.04%)	45.0 (30.71% to 60.17%)
	Specificity (%) (95% CI)	100.0 (85.69% to 100.0%)	100.0 (85.69% to 100.0%)	78.3 (58.10% to 90.34%)	100.0 (85.69% to 100.0%)	73.9 (53.53% to 87.45%)
	False negative	7.5%	12.5%	30.0%	10.0%	55.0%
	False positive	0.0%	0.0%	21.7%	0.0%	26.1%
**OvMANE1_OvMCBL02**						
ROC analysis	ROC area (AUC)	0.9793	0.9685	0.8304	0.9772	0.5158
	95% CI of AUC	0.9513 to 1.000	0.9328 to 1.000	0.6932 to 0.9676	0.9467 to 1.000	0.3677 to 0.6638
	*p*-value (against AUC = 0.5)	<0.0001	<0.0001	<0.0001	<0.0001	0.8360
Diagnostic accuracy parameter	Cutoff value	1.157	0.4275	0.0495	0.3150	0.0480
	Sensitivity (%) (95% CI)	90.0 (76.95% to 96.04%)	72.5 (57.17% to 83.89%)	90.0 (76.95% to 96.04%)	92.5 (80.14% to 97.42%)	37.5 (24.22% to 52.97%)
	Specificity (%) (95% CI)	100.0 (85.69% to 100.0%)	100.0 (85.69% to 100.0%)	82.6 (62.86% to 93.02%)	100.0 (85.69% to 100.0%)	60.8 (40.79% to 77.84%)
	False negative	10.0%	27.5%	10.0%	7.5%	62.5%
	False positive	0.0%	0.0%	17.4%	0.0%	39.2%

## Data Availability

The datasets generated during this study are available from the corresponding authors on reasonable request.
